# Smartphone App Using Reinforcement Learning for Obesity: Single-Arm Feasibility Study

**DOI:** 10.2196/77323

**Published:** 2026-02-26

**Authors:** Ken Kurisu, Yoshiharu Yamamoto, Tomohisa Aoyama, Toshimasa Yamauchi, Kazuhiro Yoshiuchi

**Affiliations:** 1 Department of Stress Sciences and Psychosomatic Medicine, Graduate School of Medicine, The University of Tokyo, 7-3-1, Hongo, Bunkyo-ku, Tokyo, 113-8655, Japan, 81 3-5800-9764; 2 Educational Physiology Laboratory, Graduate School of Education, The University of Tokyo, Tokyo, Japan; 3 Department of Diabetes and Metabolic Diseases, Graduate School of Medicine, The University of Tokyo, Tokyo, Japan

**Keywords:** cognitive behavioral therapy, ecological momentary intervention, machine learning, multiarmed bandit, obesity, reinforcement learning, smartphone app

## Abstract

**Background:**

While behavioral interventions remain an evidence-based treatment for obesity, they often require long durations and frequent sessions. To address this, we hypothesized that interventions delivered in daily life via a smartphone app combined with personalized optimization using reinforcement learning may effectively support behavior changes.

**Objective:**

This study aimed to develop and evaluate the feasibility of such an app for individuals with obesity.

**Methods:**

We developed a smartphone app to assist in setting and reviewing daily behaviors related to weight loss. On the screen on which daily behaviors were shown, the order of presentation was optimized using Thompson sampling, a multiarmed bandit algorithm. Twenty individuals with obesity used the app for 4 weeks, and the daily app use rates were quantified. Body weight and mood status were measured daily during the study, and a brief-type self-administered diet history questionnaire and the International Physical Activity Questionnaire were administered at the beginning and end of the study. Changes in these measures were evaluated using the Wilcoxon signed rank test. Furthermore, the longitudinal data collected during this study were analyzed using a linear mixed-effects model to examine factors related to the number of behaviors performed daily.

**Results:**

All 20 recruited individuals with obesity completed the 4-week study schedule. The median app use rate was 98.3% (range 76.9%‐100%). Significant improvements were observed in BMI (median at start 34.9 kg/m^2^, range 27.4-52.9; median at end 34.1 kg/m^2^, range 26.7‐51.0; *P*=.01), as well as daily energy intake and weekend sitting time. The linear mixed-effects model showed a significant association between higher preceding depressive mood levels and fewer behaviors (*P*<.001).

**Conclusions:**

The feasibility of the smartphone app using reinforcement learning for obesity was sufficient, and the potential effectiveness of the treatment was suggested. Preceding depressive mood may influence daily behaviors related to weight loss.

## Introduction

Obesity leads to diseases such as type 2 diabetes and hypertension, which further increase the risk of serious complications, including cardiovascular diseases and certain types of cancer [1]. Obesity often coexists with psychiatric disorders and has a bidirectional relationship with them [2]. Obesity reduces well-being, resulting in a vicious cycle that leads to further weight gain [3]. The prevalence of obesity is increasing worldwide, including in Japan [4], underscoring the need for effective treatment.

Current obesity treatments include glucagon-like peptide-1 receptor agonists and bariatric surgery, which cause adverse gastrointestinal effects and postoperative psychiatric comorbidities, respectively [5-7]. Behavioral interventions remain important as a low-risk and low-cost treatment [5,8], and successful long-term weight maintenance often relies on behavioral strategies [5]. Although structured cognitive behavioral therapy for obesity has been developed [9,10], the frequent and long duration of sessions burdens both participants and therapists. Furthermore, behavioral science findings indicate that reinforcers should be provided immediately after performing behaviors [11], which is challenging in outpatient settings. To address these issues, we hypothesized that ecological momentary intervention (EMI) through smartphone apps in daily life would be helpful [12,13].

In the context of EMI for obesity, tailored presentation of behavioral content may play an important role in promoting effective behavior change. In face-to-face cognitive behavioral therapy, behavior changes for diet and physical activity are tailored to individuals’ daily lifestyles and preferences [9,10]. From a technical perspective, machine learning and reinforcement learning have been widely deployed for content optimization in modern web applications and smartphone apps [14]. One study used a multiarmed bandit algorithm, an optimization technique using reinforcement learning, to suggest health behaviors [15]. The algorithm explores items with limited prior information to reduce uncertainty and simultaneously exploits items with higher estimated rewards based on accumulated data. Such optimization by multiarmed bandit algorithms is applicable for recommending daily behaviors to individuals with obesity through smartphone apps.

Numerous studies on smartphone apps and web-based interventions for obesity have been conducted, and an umbrella review suggests that they can achieve greater weight loss when combined with usual treatments [16]. Relevant studies have involved sending text messages related to physical activity or dietary intake [17-20]. However, to the best of our knowledge, no study has used an app specifically designed for setting daily behaviors with personalization using machine learning or reinforcement learning. Accordingly, such an app would be novel and could lead to more effective treatments.

Additionally, the completion of behaviors related to weight loss suggested through smartphone apps may be influenced by daily psychosocial factors. Many psychosocial factors reportedly influence the maintenance and progression of obesity [2,3,5]. Studies using ecological momentary assessments, which analyze data collected in daily life, have suggested that preceding mood status and other factors influence daily calorie intake and physical activity in individuals with obesity [21-24]. Examining the relationship between daily behaviors and preceding mood collected through a smartphone app may help improve the app.

In summary, we hypothesized that interventions in daily life delivered via smartphone apps could effectively address current challenges in behavioral interventions and that personalization through multiarmed bandit algorithms could further enhance their effectiveness. Therefore, this study aimed to develop a smartphone app with a multiarmed bandit algorithm for individuals with obesity and evaluate its feasibility. In addition, as psychosocial factors may influence the effectiveness of this intervention, the secondary aim was to examine factors influencing daily behaviors related to weight loss.

## Methods

### Ethical Considerations

This study was approved by the Institutional Review Board of the University of Tokyo (2022061NI). The research protocol was registered with the University Hospital Medical Information Network Clinical Trials Registry (UMIN000048667). All the participants provided written informed consent. All data were anonymized, and access was limited to authorized research team members only. Participants received up to JP¥ 20,000 (approximately US $150 based on the exchange rate at the time of study start) via QUO Card.

### Smartphone App

**Figure 1.**
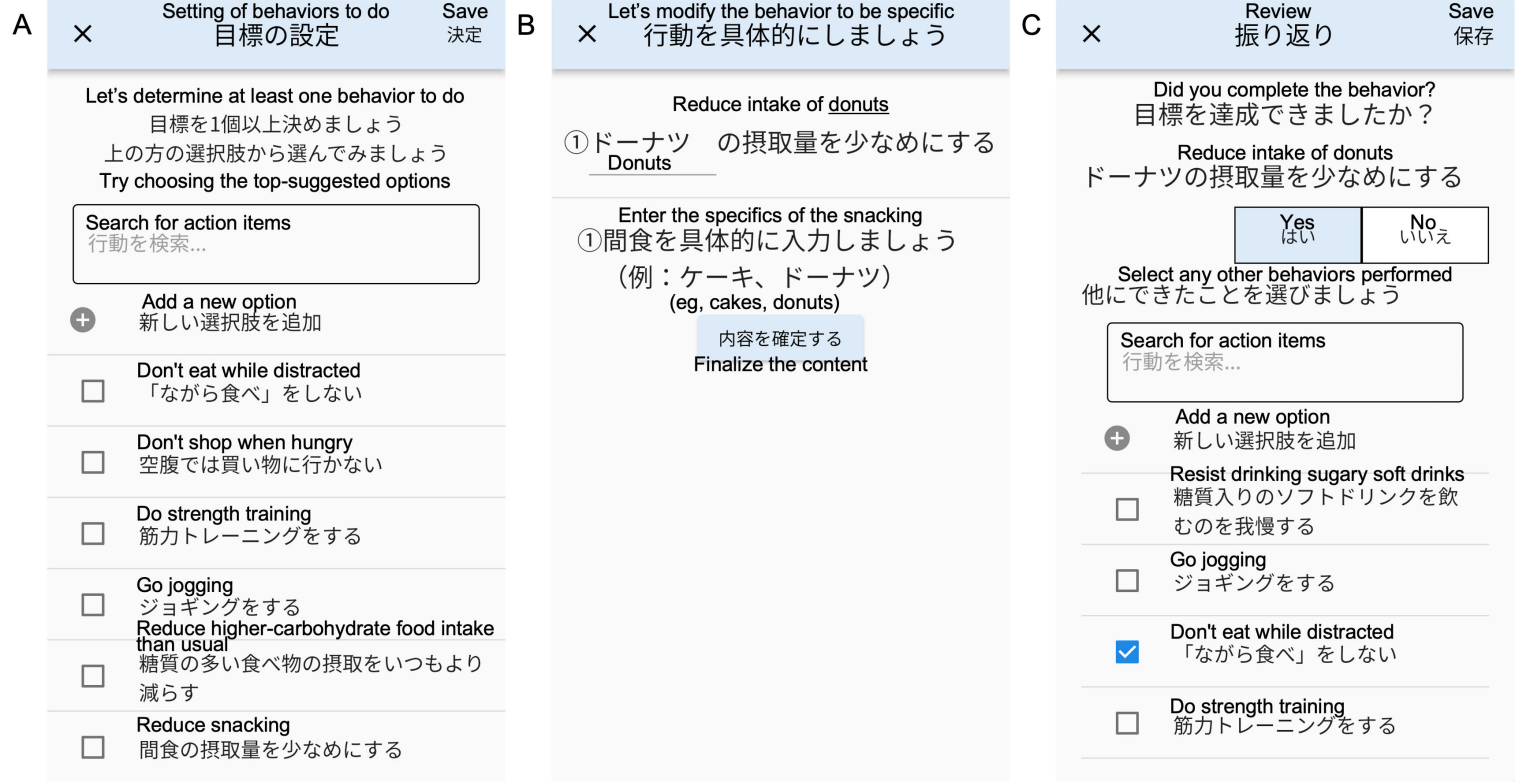
Screenshots of the app (translated from Japanese into English): (A) at the end of the day, users select behaviors to perform the following day, with on-screen guidance encouraging them to choose top-listed items suggested by a reinforcement learning algorithm; (B) selected behaviors can be specified further (eg, “Reduce snacking” to “Reduce intake of donuts”); and (C) a notification is sent at the end of the following day to record whether the selected behaviors were performed. Users can also log additional behaviors performed.

Figure 1 shows screenshots of the smartphone app we developed for obesity management. Users launch the app upon receiving a notification at the end of the day, typically in the evening at an individually preset time, and select behaviors to perform the following day (Figure 1A). The selected behaviors can be further modified for specificity (Figure 1B). The user receives reminder notifications several times the following day regarding the behaviors they have set. Upon receiving a notification at the end of the following day, users launch the app to record whether they performed the selected behaviors and log any additional behaviors (Figure 1C). Users then select the behaviors to perform the following day. The researchers prepared a behavioral list based on the cognitive behavioral therapy manual [9] and the Japanese guidelines for obesity management [4]. Each participant can also create original items individually. The app displays a complimentary message and a graphical presentation of the number of behaviors performed immediately after recording them.

The presentation order was optimized to display easily achievable, low-effort items earlier on the screen (Figure 1A). This optimization was based on the theory of cognitive behavioral therapy [9,10] and the Fogg behavior model [25], similar to a study on a smartphone app using reinforcement learning to promote health-related activities [15]. A multiarmed bandit algorithm was used, which simultaneously performs exploration (collecting data to determine which items are achievable) and exploitation (presenting achievable items based on the collected data). This has the advantage that it can be applied to even a small number of users while ensuring a diversity of suggestions [14].

We used Thompson sampling, a multiarmed bandit algorithm that does not require prior hyperparameter adjustments [14]. In this study, each behavior was treated as an independent arm and scored by a randomly sampled value of θ from the following beta distribution, where *N* is the number of times the user selects the item and *a* is the number of executions:


$$p\left(\theta |a,N\right)=\frac{\theta^{a}{\left(1-\theta \right)}^{N-a}}{\int_{0}^{1}\theta^{a}{\left(1-\theta \right)}^{N-a}d\theta }$$

When *N* is small, samples of θ from the beta distribution exhibit a large variance, and the model assigns random scores to behaviors with few selections, which promotes exploration. As *N* increases, the samples converge toward the actual achievable rate of each behavior, which promotes exploitation by assigning higher scores to behaviors with higher completion rates than to those with lower completion rates. In this study, each participant had an independent model trained using only their own behavior data. Each behavior was initialized with a uniform prior over the interval (0, 1).

The app was developed using Flutter (Google) and Firebase (Google). Reinforcement learning was implemented using Python (version 3.9; Python Software Foundation) on the Google Cloud Platform. The app was distributed exclusively to participants’ smartphones (iPhone or Android) through DeployGate (DeployGate, Inc) [26] rather than being publicly released on the Apple App Store or Google Play Store.

### Study Participants

This study included individuals diagnosed with “obesity disease” according to criteria established by the Japan Society for the Study of Obesity. This disease is defined as a BMI of ≥25 kg/m^2^ with one or more obesity-related disorders such as type 2 diabetes or visceral fat accumulation that likely causes health problems [4,27]. Individuals aged ≥18 years attending the University of Tokyo Hospital were eligible for this study. Exclusion criteria were (1) extremely severe physical or psychiatric diseases, (2) conditions for which the burden of the study would be harmful, and (3) lack of decision-making ability.

A previous study examining the feasibility of an app using a multiarmed bandit algorithm included approximately 20 participants over 3 weeks [15]. Following this, we set the study period to 4 weeks (28 days) and the sample size to 20. Eligible individuals were recruited continuously until the target sample size was achieved.

### Study Procedure

**Figure 2.**
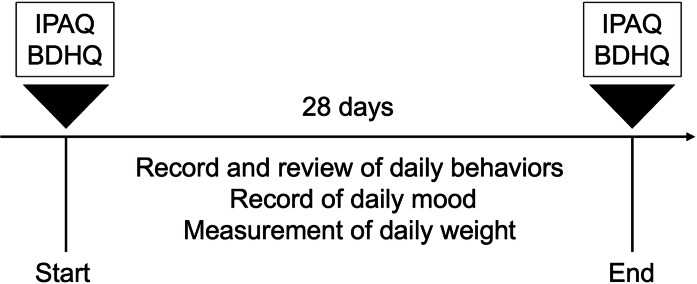
Study schedule. The study period lasted 28 days, with the International Physical Activity Questionnaire (IPAQ) and brief-type self-administered diet history questionnaire (BDHQ) measured at the start and end of the study. Daily behaviors related to weight loss, mood, and weight were recorded during the study period.

Figure 2 provides the study schedule. At the beginning of the study, the Japanese version of the International Physical Activity Questionnaire (IPAQ) long form, last–7-day version, was used to assess physical activity [28]. The brief-type self-administered diet history questionnaire (BDHQ) was used to assess nutritional intake [29]. The BDHQ consists of questions assessing the consumption frequency of 58 food and beverage items over the preceding month and estimates average daily energy intake and various nutrient intakes.

**Figure 3.**
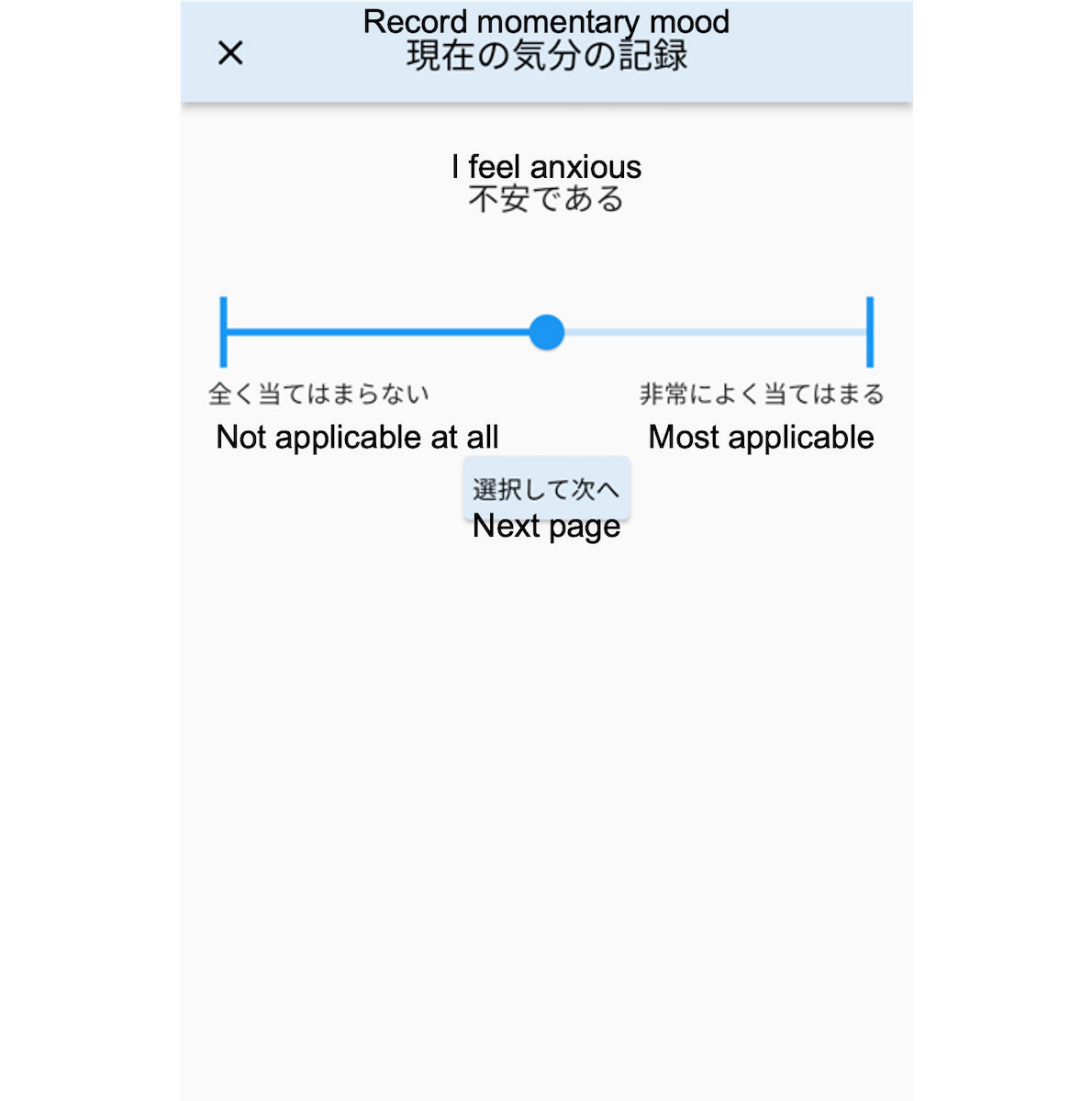
Screenshot of records of daily momentary mood status (translated from Japanese into English).

During the study, daily behaviors were recorded using the app. Participants received a notification daily at a random time within a prearranged 1-hour interval and recorded their mood status (Figure 3). Nine items from the Depression and Anxiety Mood Scale were used to calculate scores for anxiety, positive affect, and depression [30]. This was recorded on a 101-point (0‐100) visual analog scale. Additionally, we provided the participants with a body scale (HN300T2; OMRON) connected via Bluetooth to a smartphone (iPhone or Android) to record weight automatically [31]. Their weight was recorded daily upon waking.

At the end of the 4-week study, the IPAQ and the BDHQ were administered, as at the start of the study. The top 5 behaviors displayed on the app screen were evaluated on paper using a visual analog scale regarding their subjective likelihood of achievement. In addition, qualitative interviews were conducted to identify potential improvements to the app.

### Statistical Analyses

Feasibility was assessed via app use rate, defined as the percentage of days on which the behavior was recorded during the study period. Relevant EMI studies using smartphone apps for obesity or type 2 diabetes have reported recording rates of approximately 80% to 95% [20,32,33], which we set as the benchmark for feasibility.

BMI, physical activity measured using the IPAQ, and calorie and salt intake measured using the BDHQ were compared between the start and end of the study using the Wilcoxon signed rank test, with statistical significance set at .05.

These analyses were performed using R (version 4.3.1; R Foundation for Statistical Computing).

### Analysis of Predictors of Daily Behaviors for Weight Loss

In this post hoc analysis, we combined data on daily behaviors with mood status recorded within the preceding 24 hours. Only behavioral records reviewed more than 12 hours and less than 36 hours after setting were included. A linear mixed-effects model was developed using this nested longitudinal dataset, with the daily number of behaviors performed as the outcome. The outcome was defined as the sum of the preselected behaviors that were actually performed and any additional behaviors.

Level 1 variables included (1) preceding mood status (anxiety, positive affect, and depression) measured using the Depression and Anxiety Mood Scale, which was scaled from 0 to 1; (2) the difference between the weight recorded immediately before the behavior review and the weight at the start of the study; (3) the difference between the weight recorded immediately before the behavior review and the weight recorded at one earlier time point; and (4) the number of weeks, ranging from 1 to 4. Level 2 variables included (1) sex; (2) BMI at the start, centered by subtracting 35; and (3) the presence of comorbid psychiatric disorders. The inclusion of these variables and random effects was determined based on the Akaike information criterion.

R (version 4.3.1) and the package *lmerTest* (version 3.1-3) were used for this analysis.

## Results

### Feasibility Evaluation

Participants were recruited from August 2022 to July 2023, and all 20 individuals with obesity completed the 4-week study. Table 1 provides the descriptive statistics. The median app use rate, defined as the percentage of days on which behaviors were recorded, was 98.3% (range 76.9%‐100%), with a mean of 94.8% (SD 7.1%).

**Table 1.** Characteristics of the participants (N=20).

	Values
Age (years), median (range)	43 (21-66)
Sex, n (%)	
Male	9 (45)
Female	11 (55)
BMI at start (kg/m^2^), median (range)	34.9 (27.4-52.9)
Height (cm), median (range)	164.4 (151.0-184.0)
Weight at start (kg), median (range)	93.6 (70.5-158.3)
Bariatric surgery (laparoscopic sleeve gastrectomy)	
Individuals who underwent surgery, n (%)	4 (20)
Postoperative period (months), median (range)	17.6 (7.8-45.1)
Glucagon-like peptide-1 receptor agonist use	
Semaglutide, n (%)	7 (35)
Liraglutide, n (%)	1 (5)
Duration of use (months), median (range)	6.3 (0.3-154.9)
Physical comorbidities, n (%)	
Type 2 diabetes	12 (60)
Hypertension	14 (70)
Obstructive sleep apnea	10 (50)
Dyslipidemia	13 (65)
Psychiatric comorbidities, n (%)	
Depression	2 (10)
Bipolar disorder	2 (10)
Adjustment disorder	2 (10)
Sleep disorder	4 (20)
Binge eating disorder	2 (10)

### Treatment Effectiveness

Table 2 shows treatment-related results. BMI decreased significantly (median at start 34.9 kg/m^2^, range 27.4‐52.9; median at end 34.1 kg/m^2^, range 26.7‐51.0; *P*=.01). In total, 15% (3/20) of the participants gained weight during the study period, 2 of whom had undergone bariatric surgery. Significant changes were also observed in calorie intake and weekend sitting time.

**Table 2.** Changes in treatment-related measurements.

	Before the intervention (baseline), median (range)	After the intervention (4 weeks), median (range)	*P* value
BMI (kg/m^2^)	34.9 (27.4‐52.9)	34.1 (26.7‐51.0)	.01
BDHQ^a^			
Calorie intake (kcal/day)	1603 (541‐4191)	1367 (527‐2572)	.04
Salt intake (g/day)	9.0 (3.7‐21.1)	8.5 (4.4‐20.9)	.09
IPAQ^b^			
Physical activity (MET^c^ minutes/week)	2716 (99‐13566)	3413 (537‐13506)	.70
Weekday sitting (hours/day)	5 (1-14)	4 (1-13)	.06
Weekend sitting (hours/day)	6.5 (2-14)	4.5 (1-12)	.008

a BDHQ: brief-type self-administered diet history questionnaire.

b IPAQ: International Physical Activity Questionnaire.

c MET: metabolic equivalent of task.

### Usability Survey

The median number of behaviors performed over the 4 weeks, including individually created behaviors, was 11 (range 6‐27). The subjective evaluation of the achievability of the items presented at the top of the app screen, which ranged from 0 to 100, had a median score of 85 (range 58‐100).

In the qualitative interviews, the most frequently raised concern was the following (8/20, 40%):

Toward the latter part of the study period, I tended to choose only a few types of items displayed at the top and felt a sense of monotony.

This implies that the algorithm converged on a small subset of items with higher completion rates toward the end of the study, resulting in these items being repeatedly displayed at the top and predominantly selected by users.

The next most frequently raised concern was the following (6/20, 30%):

I was unable to modify some items to be more specific.

This means that, although each behavior could be further specified, as exemplified in Figure 1B, users had difficulty doing so for several items.

### Predictors of Daily Behaviors for Weight Loss

A total of 432 records collected during the study were included in the analysis. The final model developed based on the Akaike information criterion is presented below, where subscript *i* denotes each participant and subscript *j* denotes the time point. $Y_{i,j}$ is the daily number of behaviors performed, ${Depression}_{i,j}$ is the preceding depressive mood, and ${Psychiatry}_{i}$ indicates the presence of psychiatric comorbidities. Fixed and random effects are represented by γ and ζ, respectively.

Level 1:


$$Y_{i,j}=\Pi_{0,i}+\Pi_{1,i}{Depression}_{i,j}+\varepsilon_{i,j}$$

Level 2:


$$\Pi_{0,i}=\gamma_{0,0}+\zeta_{0,i}$$

$$\Pi_{1,i}=\gamma_{1,0}+\gamma_{1,1}{Psychiatry}_{i}+\zeta_{1,i}$$

The coefficients of each variable are listed in Table 3. The following can be interpreted from each coefficient: (1) a higher preceding depressive mood was associated with fewer behaviors, and (2) individuals with comorbid psychiatric disorders had smaller coefficients for preceding depressive mood.

**Table 3.** Coefficients of the linear mixed-effects model for the daily number of performed behaviors.

	Fixed effects coefficient (95% CI)	*P* value
Intercept ($\gamma_{0,0}$)	4.45 (3.47 to 5.43)	<.001
Depression ($\gamma_{1,0}$)	−1.88 (−2.87 to −0.88)	<.001
Depression × psychiatric comorbidity ($\gamma_{1,1}$)	1.59 (0.34 to 2.85)	.01

## Discussion

### Principal Results

The smartphone app developed in this study for individuals with obesity was used by 20 recruited participants over 4 weeks, with a median use rate of 98.3%. Participants’ BMI decreased significantly during the study period. Furthermore, a linear mixed-effects model based on data obtained through the app showed that a higher preceding depressive mood was associated with a reduced number of completed behaviors related to weight loss.

The median use rate was 98.3%, which exceeded the adherence rates of approximately 80% to 95% reported in relevant studies [20,32,33], indicating sufficient feasibility. Future studies should address the concerns identified in the qualitative interviews. To prevent the selection of behaviors from being monotonous, items that are consistently completed may be retired from the list. In addition, improving the clarity of item explanations may effectively address the challenges in specifying item content.

The median decrease in BMI was 2.3% over 4 weeks. The Japanese clinical guidelines recommend a 3% weight loss for individuals with a BMI of <35 kg/m^2^ and a 5% to 10% weight loss for those with a BMI of ≥35 kg/m^2^ over 3 to 6 months [4]. Accordingly, the weight loss achieved in this study appeared sufficient. Calorie intake and weekend sitting time improved, and these behavior changes may have contributed to weight loss. Thus, the developed app may be effective for weight loss.

The effect size should be interpreted with caution due to several features of this study design. Daily weight measurements were conducted throughout the study, which may have influenced weight loss [34]. In total, 15% (3/20) of the participants gained weight during the study, 2 of whom had undergone bariatric surgery, a population known to frequently experience weight regain [35]. A previous umbrella review of smartphone app and web application interventions for obesity showed a mean difference of −0.12 kg compared with face-to-face treatment and −4.32 kg compared with no treatment [16]. However, our study was a single-arm trial with a follow-up duration different from those of previous studies, making direct comparisons difficult. Therefore, further studies are warranted to more accurately determine the effect size of the app developed in this study.

Given the high ratings for the subjective likelihood of achievement of the top-suggested items, with a median of 85 on a scale from 0 to 100, the multiarmed bandit algorithm seemingly approached convergence. Qualitative interviews revealed that the participants tended to choose only a few types of items presented at the top and reported a sense of monotony, further suggesting algorithm convergence. Future studies with more accumulated data should evaluate the convergence of the algorithm and may consider applying other optimization approaches such as collaborative filtering [14]. On the basis of the Fogg behavior model [25], this study prioritized behaviors based solely on completion rates; however, future studies should also consider the behaviors’ contributions to clinical outcomes such as weight loss, along with the effort and duration required for achievement.

In the post hoc analysis, the linear mixed-effects model showed that preceding depressive mood was related to weight loss behaviors. This aligns with a previous study showing that baseline depression is associated with emotional eating and binge eating [22]. One study showed that delay discounting mediates the association between depressive symptoms and the onset of diabetes [36], which may help explain these findings. Another study suggested that individuals with higher levels of depression exhibited unhealthy behaviors such as physical inactivity [37]. On the basis of these findings, future studies may benefit from providing suggestions on the number of behaviors to select on the app and prioritizing lower-effort behaviors according to daily depression levels. The interaction coefficient of depressive mood and psychiatric comorbidity was positive, indicating that, when adjusted for depressive mood as a covariate, the number of behaviors performed was large for individuals with psychiatric diseases. This may suggest that treating psychiatric disorders positively influences weight loss behaviors; however, further studies are needed to interpret these findings.

### Limitations and Future Directions

This study has several limitations. First, the study period was short, and long-term use of the app remains unexplored. Second, the dietary and physical activity questionnaires were administered only at baseline and after the intervention, which limited assessment of temporal changes and intervention feasibility. Third, energy intake estimated using the BDHQ appeared low. Alternative assessment tools such as the Automated Self-Administered 24-hour dietary recall [38] may be preferable in future studies. Fourth, this study used weight alone without distinguishing between fat and muscle mass; this is particularly relevant for participants receiving glucagon-like peptide-1 receptor agonists or who underwent bariatric surgery. Future studies should assess body composition using tools such as bioelectrical impedance analysis. Fifth, in the optimization algorithm, the behaviors were evaluated only with regard to achievability. Their contributions to weight loss may vary and should ideally be incorporated into the algorithm. Sixth, the impact of the multiarmed bandit algorithm on optimization remains unevaluated. Seventh, the effect size for weight loss was inconclusive in this single-arm study. Finally, the analysis of the relationship between preceding depressive mood and daily behaviors used only the subjective input of behavioral execution.

Future research should improve the app based on the findings of this study and assess the treatment efficacy through a randomized controlled trial with a longer follow-up period. Additionally, future studies should assess the contribution of optimization to weight loss using a randomized design that compares optimized and nonoptimized interventions. Furthermore, although this study used an item-fixed measurement based on classic test theory, future research could use computer-adaptive tests [39,40] to measure mood status more efficiently.

### Conclusions

The smartphone app for obesity developed in this study demonstrated sufficient feasibility, and its potential effectiveness for weight loss was suggested. Data obtained through this app indicate that a preceding depressive mood may influence daily behaviors related to weight loss.

## Abbreviations

BDHQ: brief-type self-administered diet history questionnaire
EMI: ecological momentary intervention
IPAQ: International Physical Activity Questionnaire
